# Single Nucleotide Polymorphisms of MicroRNA Processing Machinery Genes and Outcome of Hepatocellular Carcinoma

**DOI:** 10.1371/journal.pone.0092791

**Published:** 2014-03-27

**Authors:** Shuang Liu, Jie An, Jianhong Lin, Yanli Liu, Lidao Bao, Wen Zhang, Jian-Jun Zhao

**Affiliations:** 1 Department of Pathology, Bethune International Peace Hospital, Shijiazhuang, China; 2 Medical Oncology, Dana-Farber Cancer Institute, Harvard Medical School, Boston, Massachusetts, United States of America; Nanjing Medical University, China

## Abstract

MicroRNA (miRNA)-related single nucleotide polymorphisms (miR-SNPs) can affect cancer development, treatment efficacy and patients prognosis. We examined 6 miR-SNPs in miRNA processing machinery genes including exportin 5 (XPO5) (rs11077), Ran-GTPase (RAN) (rs14035), Dicer (rs3742330), Trinucleotide Repeat Containing 6B (TNRC6B) (rs9623117), GEMIN3 (rs197412), GEMIN4 (rs2740348) in 108 surgically resected HCC patients and evaluated the impact of these miR-SNPs on HCC outcome. Among the 6 SNPs, only the A/A genotype of rs11077 located in XPO5 3′UTR was identified to associated independently with worse survival in HCC patients by multivariate analysis with relative risk, 0.395; 95% CI, 0.167–0.933; p = 0.034. This is the first study reporting that polymorphisms related to miRSNPs have prognostic value in hepatocellular carcinoma and identify the A/A genotype of rs11077 SNP site located in XPO5 3′UTR can help to predict worse prognosis in patients.

## Introduction

Hepatocellular carcinoma (HCC) is the fifth most common cancer and is responsible for more than half a million deaths each year, which makes it the third leading cause of cancer deaths worldwide [1]. The severity of HCC and the lack of effective treatment strategies make the disease a major challenge. This disease is strongly associated with several risk factors, including chronic hepatitis B virus (HBV) infection, chronic hepatitis C virus (HCV) infection, and alcohol abuse [2]. The incidence of HCC has increased steeply in Asia and Africa, where HBV and HCV infections are more prevalent than in other continents. HBV infection is a challenging health issue in China, where approximately 93 million people are HBV carriers and 30 million have chronic hepatitis B [2], [3]. Alcohol abuse is also increasing in China, where ∼6.6% of males and 0.1% of females in the population have been diagnosed with alcoholism [4]. Many of these people develop liver disease, such as alcoholic hepatitis and cirrhosis, which make them susceptible to HCC. Despite improved clinical detection methods and therapies, the prognosis of post-operative HCC patients is still poor, due to a high recurrence rate. While the molecular mechanism of HCC carcinogenesis is still not fully understood, there are many prognostic factors and predictors of recurrence associated with the disease, including tumour size, tumour quantity, cell differentiation, venous invasion and degree of inflammation [5]–[9].

MicroRNAs (miRNAs) are RNA molecules with lengths of ∼22 nucleotides that act as posttranscriptional regulators of mRNA expression [10], [11]. Over 1000 miRNAs have been identified in humans, and these miRNAs are responsible for regulating the expression of at least 30% of protein-coding genes. A growing body of evidence suggests that miRNAs play important roles in a broad range of biological processes, such as embryonic development, cellular differentiation, proliferation, apoptosis, cancer development and insulin secretion [10], [11]. In the miRNA processing, long primary transcripts of miRNAs (pri-miRNAs) are processed in nucleus by the RNase III Drosha, transported to the cytoplasm by the nuclear transport factor exportin-5 (XPO5) and Ran-GTPase (RAN). In the cytoplasm, RNase III Dicer and transactivation-responsive RNA-binding protein (TRBP) mediate pre-miRNAs processing to release a 21-bp dsRNA, the RNA-induced silencing complex (RISC) including GEMIN3 and GEMIN4 will select one strand as the mature miRNA and guide mature miRNAs to their target mRNA sites [10], [12]–[16]. MiRNA related single nucleotide polymorphisms (miR-SNPs), defined as single nucleotide polymorphisms (SNPs) in miRNA genes, miRNA binding site and miRNA processing machinery, can modulate miRNA and targeted genes expression so as to affect cancer development, therapeutic efficacy and patient's prognosis [16]–[19].

In the present study, we genotyped 6 miR-SNPs in miRNA processing machinery genes including XPO5 (rs11077), RAN (rs14035), Dicer (rs3742330), TNRC6B (rs9623117), GEMIN3 (rs197412), GEMIN4 (rs2740348) in 108 surgically treated HCC patients and evaluated the impact of these miR-SNPs on HCC outcome. The post-operated HCC patients enrolled in this study were followed up with regular visit by laboratory tests combined with ultrasound, contrast-enhanced computed tomography (CT) or contrast-enhanced magnetic resonance imaging (MRI). A miR-SNP in the 3′UTR region of XPO5 was found to be an independent prognostic marker for HCC outcomes.

## Materials and Methods

### Tissue specimens and DNA extraction

Blood samples were collected at the Bethune International Peace Hospital from 108 HCC patients who underwent tumour resection in the Department of Hepatobiliary Surgery between 2003 and 2008 in accordance with Bethune International Peace Hospital Review Board approval, and informed consent performed in compliance with the Declaration of Helsinki. All participants provided their written informed consent to participate in this study. A total of 114 patients enrolled in this study were reviewed every 3 months for 3 years. Six patients were lost during follow-up: one HBV-HCC patient in the first year, one HCV-HCC and two HBV-HCC patients in the second year and one HCV-HCC and one HBV-HCC patients in the third year. The remaining 108 patients, including 87 HBV-HCC patients, 12 HCV-HCC patients and 9 alcohol-related HCC patients, were assessed. None of these patients received adjuvant chemotherapy or radiation therapy following HCC resection. Blood was also collected from 80 healthy controls. Genomic DNA was extracted immediately with a Wizard Genomic DNA extraction kit (Promega, Madison, WI). All procedures were supervised and approved by the hospital's Human Tissue Research Committee.

### Genotyping of miR-SNPs

The miR-SNP of the miRNA processing genes including XPO5 (rs11077), RAN (rs14035), Dicer(rs3742330), TNRC6B(rs9623117), GEMIN3(rs197412), GEMIN4(rs2740348) were genotyped using the Polymerase Chain Reaction - Ligase Detection Reaction (PCR-LDR) assay with the forward and reverse primers to amplify the DNA fragments flanking miR-SNPs using the sequence in the NCBI SNP database (http://www.ncbi.nlm.nih.gov/snp/). Polymerase Chain Reaction (PCR) was performed using a PCR Master Mix Kit according to the manufacturer's instructions (Promega, Madison, WI). The ligation was performed using the different probes matched to the miR-SNPs, and the ligated products were separated using the ABI PRISM Genetic Analyzer 3730XL (Applied Biosystems, Foster City, CA). Polymorphisms were confirmed based on the length difference of ligated products. All the primers and probes were listed in Table 1.

**Table 1:** All the primers and probes used for genotyping of miR-SNPs.

Gene	rs NCBI	Primer sequence	Probe sequence
**XPO5**	rs11077 (A/C)	F GAATCTGGTCACCTGATGGGA	S1 GTACCTCCAAGGACCAGGGCTGGGA
		R GTGCCTGAGTGGACCTTGAG	S2 TTTGTACCTCCAAGGACCAGGGCTGGGC
			S3 AGTCTTTAGTGCTAACATCCCCTTT
**RAN**	rs14035 (C/T)	F GCACTTGCTCAAAATCTGTGA	S1 TTTTAGTAATCATGTTTTAATGTAGAACC
		R TAACAGCAAGAATTCCCAACC	S2 TTTTTTTAGTAATCATGTTTTAATGTAGAACT
			S3 TCAAACAGGATGGAACATCAGTGGATTT
**GEMIN4**	rs2740348 (G/C)	F TTGCCTCTGAGAAGAAGTGG	S1 TTTTTTTTGGGAGTAACAGGGCCCTCTTCCGAC
		R GACTCAGGGATGGCTCTGTC	
			S2 TTTTTTTTTTTGGGAGTAACAGGGCCCTCTTCCGAG
			S3 AGCCAGACTTGGTGTTGAGGCTGCTTTTTTT
**TNRC6B**	rs9623117 (C/T)	F TTTCTGTCTCCTCCTATCCAT	S1 TCTCCCTGTTACTCTTAAGTAGTGC
		R CATTAGTTTAGCCAACAAGGT	S2 TTTTCTCCCTGTTACTCTTAAGTAGTGT
			S3 CTCCTTTCCCCATCCACCCCATCTC
**GEMIN3**	rs197412 (T/C)	F TAGAGAAACCTGTGGAAATCA	S1 TTTTATGGTTTTGTGAGAAATAAAGTTAC
		R GAAGAGGTTCTTGAGCTGTAA	S2 TTTTTTTATGGTTTTGTGAGAAATAAAGTTAT
			S3 TGAACAGAGAGTCCCTGTGTTGGCATTT
**Dicer1**	rs3742330 (A/G)	F AAAGGTATCAAGGTCTCAGTTTG	S1 TTTTTTTTTTCAATCTTGTGTAAAGGGATTAGA
		R CTGCAGAGGATCACTGGAATC	S2 TTTTTTTTTTTTTCAATCTTGTGTAAAGGGATTAGG
			S3 CACCCTAACAGAGCAAGATCCAATATTTTTT

F:represents forwrad primer for PCR.

R:represents reverse primer for PCR.

S1 and S2 represent probes match to different alelle of the SNP.

S3 represents probes downstream of the SNP.

### Statistical analysis

Statistical analyses were performed using the SPSS 18.0 software package (SPSS Company, Chicago, IL). Overall survival (OS) was calculated from the time of operation to last follow-up or death. Survival curves were calculated using the Kaplan-Meier method, and comparisons between the curves were analyzed using the log-rank test. Multivariate survival analysis was performed using a Cox proportional hazards model. A p value of <0.05 was considered statistically significant.

## Results

### Correlation between clinical characteristics of HCC patients and overall survival

The relationship between the overall survival during the 3-year follow-up and patients' clinical characteristics was analysed using the Kaplan–Meier method and the log-rank test. No differences were detected in overall survival time among HBV-HCC, HCV-HCC and alcohol-related HCC patients; therefore, we assessed them together in further analyses. Sex and age were not statistically significant predictors of post-operative survival time; however, tumor size, tumor stage, child classification, portal vein thrombosis and rs11077 SNP site located in XPO5 3′UTR were correlated with survival time in these patients (Table 2).

**Table 2:** Univariate analysis of clinical characteristics associated with post-operational survival with HCC by log-rank test.

Characteristics		No.cases	3 years survival rate (%)	*p* value
Gender	Male	88	33.0	0.052
	Female	20	15.0	
Age(years)	≤55	56	34.6	0.086
	>55	52	25.0	
Child classification	A	94	34.0	0.014
	B	14	0.0	
Portal vein thrombosis	No	92	32.6	0.003
	Yes	16	12.5	
Size of the tumor (diameter/cm)	≤5	44	47.7	0.004
	>5	64	17.2	
TNM classification	I	54	44.4	0.000
	II	43	16.3	
	III	11	9.1	
Rs11077	AA	93	24.7	0.010
	AC+CC	15	60.0	

### Association of XPO5 polymorphisms with HCC outcome

**Figure 1:**
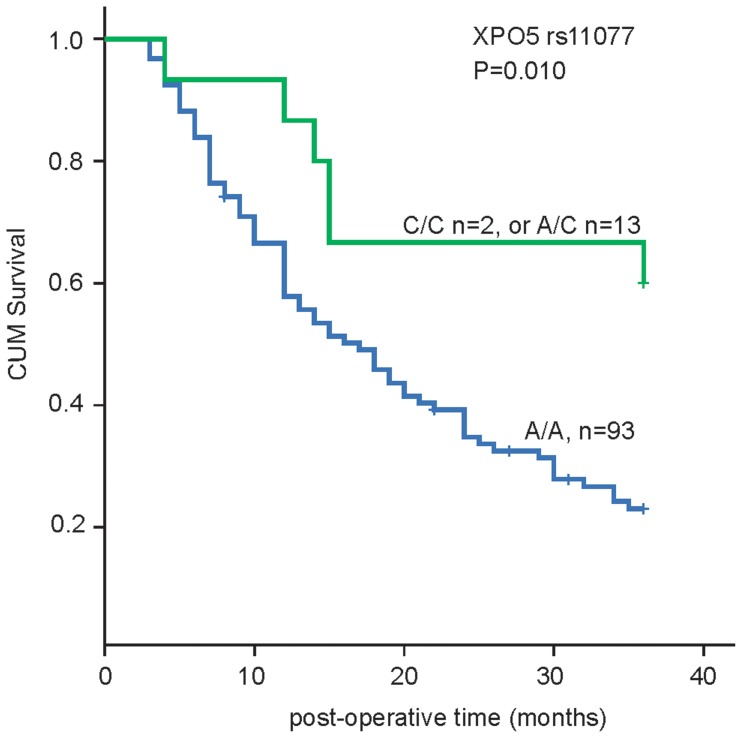
Genotype of rs11077 SNP site in XPO5 and its association with HCC survival. rs11077 SNP site in XPO5 (C/C: SNPs, A/C: heterozygous SNP, A/A:WT). Cum =  cumulative.

We genotyped these 6 miR-SNP of miRNA processing machinery genes including XPO5 (rs11077), RAN (rs14035), Dicer (rs3742330), TNRC6B (rs9623117), GEMIN3 (rs197412), GEMIN4 (rs2740348) in 108 HCC patients and 80 healthy controls, no statistically significant association (p<0.05) between patients and healthy controls was detected (data not shown). We subsequently evaluated their association for post-operational survival with Kplan-Meier methods. Among 6 SNPs, only rs11077 SNP site of XPO5 genes had prognostic impact on post-operational survival of HCC with log-rank test analysis, the three-year survival rate of A/A, and A/C+C/C ge patients were 24.7% and 60% respectively. The patients with A/A genotype of XPO5 rs11077 SNP site show a shorter overall survival compared with that of A/C+C/C genotypes (p = 0.010) as shown in Figure 1.

We then performed multivariate analysis with the Cox proportional hazards model for HCC outcome associated clinical characteristics and rs11077 SNP genotypes in these patients. As shown in Table 3, the rs11077 SNP was identified as an independent predictor of HCC outcome (relative risk, 0.395; 95% CI, 0.167–0.933; p = 0.034). Tumor stage, Child classification, but not portal vein thrombosis and size of tumor, were identified as independent predictive factors for HCC outcome. These data further demonstrated that the A/A genotype of rs11077 located in XPO5 3′UTR was associated with worse survival in HCC patients.

**Table 3:** Multivariate analysis of prognostic factors associated with HCC survival.

Factors	Relative risk	95%C.I.	P value
Child classification	1.915	1.067–3.436	0.030
TNM classification	1.525	1.023–2.274	0.038
Rs11077	0.395	0.167–0.933	0.034
Portal vein thrombosis	1.724	0.880–3.375	0.112
Size of the tumor	1.371	0.815–2.306	0.235

## Discussion

In the present study, we report for the first time that miR-SNPs have predictive value on post-operational survival of HCC patients. The miR-SNP in miRNA processing machinery genes of XPO5 are involved in the prognosis of HCC outcome. XPO5 is found in the nuclear membrane and mediates the transport of pre-miRNA between the nucleic and cytoplasmic compartments so as to adjust the whole miRNA expression level. Knock down XPO5 expression leads to reduced miRNA levels [20]. A mutated and inactive XPO5 resulted in reduced miRNA processing and decreased miRNA target inhibition, the restored XPO5 seemed as a tumor suppressor to reverse the impaired export of pre-miRNA in colon cancer [21]. The miR-SNP of rs11077 of XPO5 has been identified for its association with the cancer risk of esophageal cancer as well as the outcome of non-small-cell lung cancer and multiple myeloma [16]–[19], [22]. Nevertheless, the mechanism how this SNP modified the HCC survival remain unclear, this SNP located in 3′UTR of XPO5 might affect mRNA stability and associated with alter expression of XPO5. The altered XPO5 expression may affect the miRNAs as a whole, leading to overall suppression of miRNA expression profiles thereby mediates the HCC survival. The fact that rs11077 C/C genotype show association with reduced Renilla expression in a Renilla luciferase 3′UTR reporter system implies that this SNP could modify XPO5 expression so as to result in overall expression of miRNA in multiple myeloma cells [18]. Even though our results were concordant with the previous studies in non-small-cell lung cancer and multiple myeloma that A/C or C/C genotype of rs11077 associated with better outcome. Given the differential cell of origin for cancers and differential cell type specificity of miRNA transcriptomes, it is reasonable to assume that the effects of miR-SNP will be modulated in a cell type-specific manner. The role of XPO5 in hepatocellular cancer and the noncoding RNA including miRNA binding at this SNP site to mediate XPO5 expression need to be further investigated.

In summary, this is the first report investigating miRSNPs involved in the miRNA network in liver cancer. A polymorphism in the binding site for diverse miRNA clusters in XPO5 was associated with a significantly OS in hepatocellular carcinoma patients. MiRSNPs emerged as new promising markers for disease progression in cancer. Although miR-SNP studies for miRNA processing machinery genes are still preliminary, our results are encouraging, as they indicate that miR-SNPs could be used as cancer prognosis marker. However, the results from this study require validation in another larger HCC cohort study and in laboratory-based functional studies. MicroRNAs have been emphasised as a key factor in patients' susceptibility to therapeutic response in many complex diseases, including cancer [23]. The analysis of genetic polymorphisms in miRNA processing genes may help to identify patient subgroups with poor prognoses and may, accordingly, help to refine therapeutic decisions regarding HCC patients.
